# Effectiveness of Mobile Health–Based Exercise Interventions for Patients with Peripheral Artery Disease: Systematic Review and Meta-Analysis

**DOI:** 10.2196/24080

**Published:** 2021-02-15

**Authors:** Mihui Kim, Changhwan Kim, Eunkyo Kim, Mona Choi

**Affiliations:** 1 College of Nursing and Brain Korea 21 FOUR Project, Yonsei University Seoul Republic of Korea; 2 Department of Critical Care Nursing Samsung Medical Center Seoul Republic of Korea; 3 College of Nursing and Mo-Im Kim Nursing Research Institute Yonsei University Seoul Republic of Korea; 4 Yonsei Evidence Based Nursing Centre of Korea A JBI Affiliated Group Seoul Republic of Korea

**Keywords:** peripheral artery disease, mobile health, exercise, adherence, meta-analysis

## Abstract

**Background:**

Peripheral artery disease (PAD) affects over 236 million people worldwide, and exercise interventions are commonly used to alleviate symptoms of this condition. However, no previous systematic review has evaluated the effects of mobile health (mHealth)–based exercise interventions for patients with PAD.

**Objective:**

This study aimed to assess the effect of mHealth-based exercise interventions on walking performance, functional status, and quality of life in patients with PAD.

**Methods:**

A systematic review and meta-analysis were conducted. We searched in seven databases to identify randomized controlled trials of patients with PAD published in English up to December 4, 2020. Studies were included if patients participated in mHealth-based exercise interventions and were assessed for walking performance. We analyzed pooled effect size on walking performance, functional status, and quality of life based on the standardized mean differences between groups.

**Results:**

A total of seven studies were selected for the systematic review, and six studies were included in the meta-analysis. The duration of interventions in the included studies was 12 to 48 weeks. In the pooled analysis, when compared with the control groups, the mHealth-based exercise intervention groups were associated with significant improvements in pain-free walking (95% CI 0.13-0.88), maximal walking (95% CI 0.03-0.87), 6-minute walk test (6MWT) distance (95% CI 0.59-1.24), and walking distance (95% CI 0.02-0.49). However, benefits of the interventions on walking speed, stair-climbing ability, and quality of life were not observed.

**Conclusions:**

mHealth-based exercise interventions for patients with PAD were beneficial for improving pain-free walking, maximal walking, and 6MWT distance. We found that exercise interventions using mHealth are an important strategy for improving the exercise effectiveness and adherence rate of patients with PAD. Future studies should consider the use of various and suitable functions of mHealth that can increase the adherence rates and improve the effectiveness of exercise.

## Introduction

Peripheral artery disease (PAD) is a major cardiovascular disease characterized by limitations to arterial blood flow in the lower extremities (commonly due to atherosclerosis) and ischemia that can induce walking impairments [1,2]. PAD affects the lower extremities more commonly than the upper extremities and may present as intermittent claudication, atypical leg symptoms, critical limb ischemia, and functional impairments [1,3]. Regardless of the presence of symptoms, however, patients with PAD are at a significant risk of cardiovascular morbidity and mortality [2].

PAD is estimated to affect over 236 million people worldwide, and the prevalence increases steeply with age [4,5]. Given the increases in the pace of population aging in many countries, PAD prevalence can be expected to increase further [5].

Structured exercise programs are an important therapy for patients with PAD and can be administered in the form of supervised exercise therapy (SET) or structured community- or home-based exercise therapy (HBET) [1,3]. SET is directly supervised by health care providers in hospital or outpatient facilities, and HBET is self-exercise under the guidance of health care providers in a personal setting [3]. Structured exercise programs have been found to improve walking performance, functional status, and health-related quality of life (QoL) and also to prevent functional decline and mobility loss [3,6]. However, these benefits are most effective when patients actively and comprehensively participate in the interventions [7,8].

According to previous systematic reviews, 30.3% of the patients who participate in exercise interventions show incomplete adherence, mainly due to a lack of motivation, health reasons, patient choice, and a lack of results [6,9]. Adherence to exercise interventions is directly related to the likelihood of a participant changing or maintaining his/her health behaviors [10]. Therefore, it is necessary to find strategies for improving adherence rates to promote behavior changes.

Data-driven approaches involving the use of mobile devices such as mobile phones and wireless devices in exercise interventions have been shown to be effective for improving health outcomes [10,11]. The mobile health (mHealth) approach facilitates extensive supervision and the monitoring of patients without requiring an increase in human resources [12]. In particular, the provision of interventions based on mHealth technologies allows health care providers to provide real-time advice related to therapy and to monitor symptoms and problems without any restrictions on location [13,14]. Thus, well-designed mHealth-based interventions can be used to provide health education and promote behavior changes and have the potential to improve exercise adherence [10,12].

Previous systematic reviews on patients with PAD have mainly focused on the effects of SET, HBET, and endovascular revascularization [15-20]. In addition, some studies have sought to identify more efficient methods of PAD screening and the factors that influence participation in physical activity [21,22]. However, existing reviews have not evaluated the effects of mHealth-based exercise interventions on patients with PAD. To guide the development of future exercise interventions, there is a need for evidence regarding mHealth-based exercise interventions. Thus, in this study, we conducted a systematic review and meta-analysis with a particular focus on the effects of mHealth-based exercise interventions on walking performance, functional status, and QoL in patients with PAD. In addition, adherence rates in exercise interventions and the applied mHealth functions were investigated.

## Methods

The systematic review and meta-analysis were performed in accordance with the Preferred Reporting Items for Systematic Reviews and Meta-Analyses (PRISMA) guidelines (Multimedia Appendix 1). The protocol for this study is registered with PROSPERO (registration number: CRD 42020191744).

### Systematic Search Strategy

The systematic literature search was designed to identify randomized controlled trials (RCTs) of patients with PAD that were published in the English language in peer-reviewed journals up to December 4, 2020. We performed searches in the following seven databases: PubMed, CINAHL, Cochrane CENTRAL, EMBASE, IEEE Xplore Digital Library, Web of Science, and Scopus. We used a combination of keywords and controlled vocabulary terms such as MeSH and Emtree subject headings using Boolean operators, and this was followed by consultation with a professional medical librarian (Multimedia Appendix 2).

### Inclusion and Exclusion Criteria

Studies that met the following inclusion criteria were selected: (1) included adult patients with PAD; (2) conducted mHealth-based exercise interventions, such as with mobile phones, wearable devices, and activity trackers; (3) reported walking performance comparing an mHealth intervention group with a control group; and (4) used an RCT design. We excluded studies with the following criteria: (1) not published in English; and (2) not an original article.

### Study Selection

Based on the search strategy, studies were extracted from the seven databases and duplicates were removed. Two authors (MK and EK) independently screened the titles and abstracts of the remaining studies to determine their eligibility for the study based on the inclusion and exclusion criteria. The full texts of the selected studies were subsequently assessed and the final studies to be included in the analysis were selected. Thereafter, we manually searched the reference lists of all included studies for additional relevant studies. In instances where there were disagreements regarding decisions, a third author (CK) participated in discussions until a consensus was reached, and the resultant decision was verified by the fourth author (MC).

### Data Extraction

Two authors (MK and EK) independently extracted the data elements from the studies included in the final analysis. The data sheet contained fields for first author, year of publication, country, participants, indication, sample size, age of participants, study duration, intervention, comparator, follow-up assessment, and outcomes. If some elements of the desired data were not reported in a study, we contacted the corresponding author of the study in an attempt to obtain these data. When a study included multiple control arms, we utilized the control arm that most closely matched the intervention, such as a group that received advice regarding walking and a group that was administered a light-resistance exercise program.

### Quality Assessment

Two authors (MK and EK) independently assessed the risk of bias in accordance with the Cochrane Collaboration’s risk of bias tool, which focuses on seven domains: random sequence generation, allocation concealment, blinding of participants and personnel, blinding of outcome assessment, incomplete outcome data, selective reporting, and other sources of bias (which were scored as high, low, or unclear) [23]. In the case of disagreement, a third person (CK) participated in the discussion to resolve the disagreement. The results of the risk of bias assessment were inputted into the software Review Manager (RevMan) version 5.4 (The Cochrane Collaboration, 2020) to visually represent the results.

### Data Synthesis and Statistical Analysis

The meta-analysis was conducted to compare the standardized mean difference in walking performance, functional status, and QoL pre- and postintervention in patients who were randomly allocated to mHealth intervention groups or control groups. We analyzed continuous data by computing the mean and SD, and the standardized mean differences (SMDs) of the outcome variables were calculated using Hedges *g*, which was weighted according to the sample sizes in the studies [24]. When an original study did not provide SD values, we calculated them using reported 95% CI, range, and sample size according to the guidelines [24] and estimating formulae [24,25].

The SD values were calculated using the following formulae:


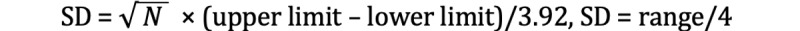


In the meta-analysis, the change-from-baseline value of the outcome variables was used, and if there were no change values, the postintervention values were used for analysis [24]. When such data were not available from the original study, we contacted the corresponding authors to request the relevant data. In a few cases, the data remained unavailable, so we excluded one study from the functional status analysis [26] and two studies from the QoL analysis [26,27] in the meta-analysis.

We assessed heterogeneity using two methods. First, we qualitatively performed a clinical judgment of differences in study populations and the follow-up durations of each study. Second, we performed a quantitative assessment, determining statistical heterogeneity using Cochrane Q (χ^2^ test) and *I*^2^ statistics after visual inspection of forest plots [24]. We considered heterogeneity to be substantial if the *I*^2^ value was greater than 75% or if the P value of the χ^2^ test was less than 0.1. We utilized a random-effects model to calculate the effect size when the included studies were heterogeneous [24]. We used a fixed-effect model, as the number of studies included was small, which presented a risk that the estimation of variance between the studies would be inaccurate [28].

In the meta-analysis of walking performance, the outcomes measured at baseline and at 12 weeks were used for analysis to reduce the heterogeneity of the study periods. When an original study did not measure outcomes at 12 weeks, we did not include it in the pooled analysis [29]. Four of the five studies used change-from-baseline values [26,27,30,31], and one study used postintervention values because the mean change could not be calculated [32] according to the Cochrane handbook for systematic reviews of interventions [24]. Pain-free walking was measured based on claudication onset time or claudication distance, which was defined as the moment the patient wished to stop walking as a result of claudication [26,27,30,31]. Maximal walking was measured using peak walking time or maximum walking distance, which was defined as the moment the patient was forced to stop walking as a result of reaching maximal claudication level [26,27,30,31]. The 6-minute walk test (6MWT) was used to measure the distance that the participants walked in the hallway for 6 minutes [29,32].

Sensitivity analysis was conducted to assess the influence of one single study being removed on the overall effect size. We did not use funnel plots for assessing publication bias, as the number of studies included was less than 10 [33]. Where statistical pooling was not appropriate, the findings were synthesized narratively. All analyses were conducted using RStudio (version 1.3.1056; RStudio, PBC).

## Results

### Study Selection

**Figure 1:**
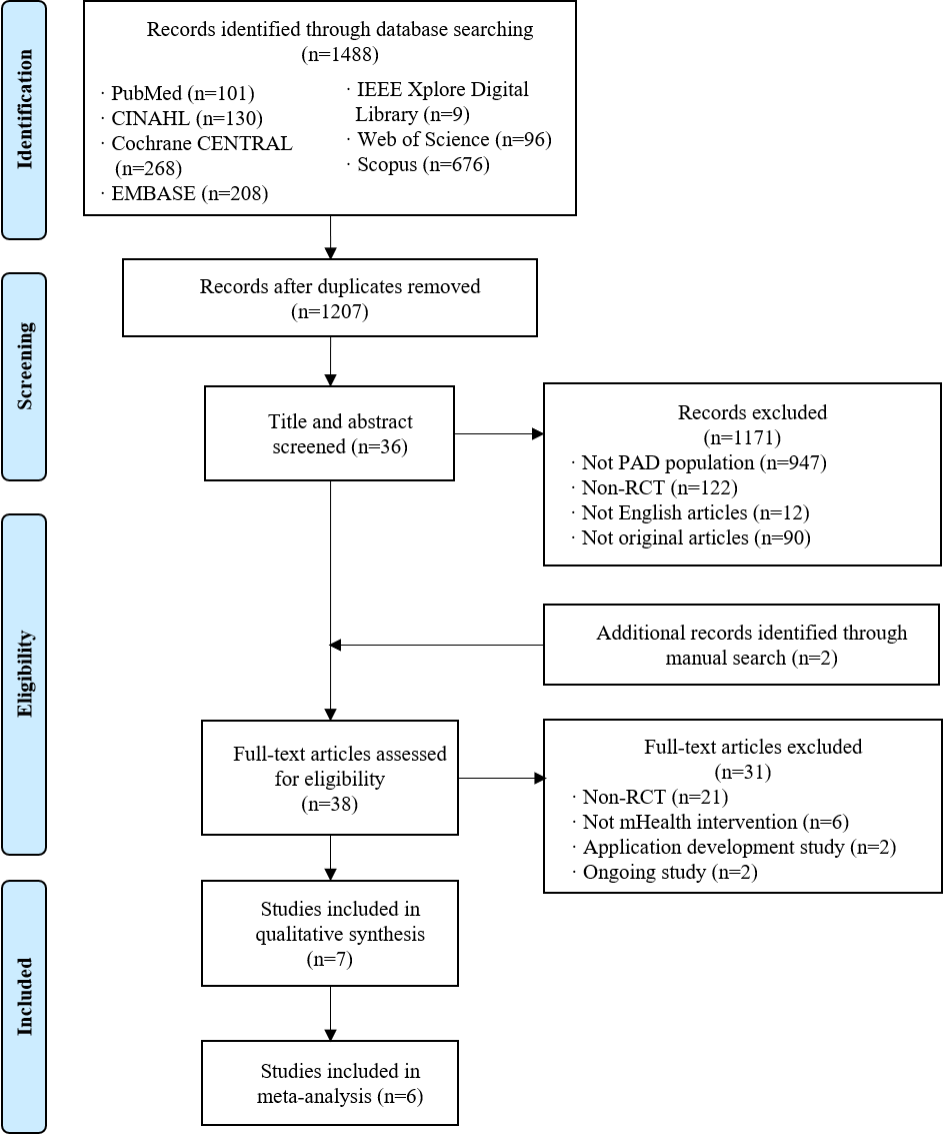
PRISMA (Preferred Reporting Items for Systematic Reviews and Meta-Analyses) flow chart of the study selection process. mHealth: mobile health; PAD: peripheral artery disease; RCT: randomized controlled trial.

Figure 1 shows our search process and the results obtained through our search strategy in a PRISMA flow chart.

We identified a total of 1488 articles from the search of seven databases. After we eliminated duplicates, 1207 articles remained. Of these remaining articles, a further 1171 were excluded based on screening of their titles and abstracts. Two were included after manual searches, and an additional 31 were excluded after full-text readings. Finally, seven studies met the inclusion criteria and were included in the review, and six of them were included in the meta-analysis. One study was excluded from the meta-analysis because it applied mHealth strategies to the SET group, in contrast to the other studies, which applied mHealth strategies to the HBET group; including this study would have resulted in difficulties merging the effect sizes [34].

### Study Characteristics

The characteristics of the seven included studies are presented in Table 1.

One study was a pilot RCT [31], and the other studies were RCTs [26,27,29,30,32,34]. Of the studies, five were undertaken in the United States [26,27,29,30,32], one in the United Kingdom [31], and one in the Netherlands [34]. All participants were diagnosed with PAD; five studies included patients with symptomatic PAD [26,27,30,31,34], one study included patients with symptomatic or asymptomatic PAD [29], and one study included patients with asymptomatic PAD [32]. The number of participants varied from 19 to 300, and the mean age of the participants was over 65 years. Of the studies, six applied mHealth strategies in HBET [26,27,29-32] and one study applied mHealth strategies in SET [34]. Regarding the total duration of the studies, four studies were performed for 12 weeks [26,27,30,32], one study was performed for 36 weeks [29], and two studies were performed for 48 weeks [31,34].

**Table 1:** Characteristics of the included studies (N=7).

Study	Country	Indication of participants	Sample size (n)	Age of participants (years), mean (SD)	Study duration (weeks)
[34]	Netherlands	Fontaine stage II	mHealth^a^-based SET^b^ group: 90; SET group: 109; control group: 101	mHealth-based SET group: 65.6 (10.5); SET group: 66.1 (9.0); control group: 66.9 (8.6)	48
[31]	United Kingdom	Intermittent calf claudication	mHealth-based HBET^c^ group: 20; control group: 17	69.1 (10.4)	48
[30]	United States	Exercise limited by claudication and resting ankle brachial index <0.90	mHealth-based HBET group: 10; control group: 9	69.4 (8.4)	12
[26]	United States	Symptomatic PAD^d^	mHealth-based HBET group: 60; SET group: 60; light-resistance exercise program group: 60	65 (9)	12
[27]	United States	Intermittent claudication	mHealth-based HBET group: 40; SET group: 40; control group: 39	mHealth-based HBET group: 65 (11); SET group: 66 (12); control group: 65 (10)	12
[29]	United States	Regardless of symptoms, ankle brachial index <0.9	mHealth-based HBET group: 97; control group: 101	mHealth-based HBET group: 70.1 (10.6); control group: 70.4 (10.1)	36
[32]	United States	Asymptomatic PAD and resting ankle brachial index <0.9	mHealth-based HBET group: 19; control group: 19	mHealth-based HBET group: 68 (7.5); control group: 68 (10.6)	12

^a^mHealth: mobile health.

^b^SET: supervised exercise therapy.

^c^HBET: home-based exercise therapy.

^d^PAD: peripheral artery disease.

### Characteristics of Study Outcomes

All studies included comparators, and they are presented with the intervention methods and outcomes in Table 2.

One study did not administer any intervention to the comparators [29]; four studies provided advice on walking exercises, brochures, a book, or a related video series [27,30,32,34]; one study provided a light-resistance exercise program [26]; and one study did not report any intervention [31]. In all studies, the outcome variables were measured through objective indicators (walking performance), and the subjective indicators of functional status and QoL were measured in five studies [26,27,29,31,34]. Walking performance was measured through a treadmill test or 6MWT at baseline and at the end of the study or at certain time points during the study. In six studies, the results of the walking performance tests showed that the mHealth group significantly improved [26,27,30-32,34]. Functional status was measured using a PAD-specific measure of the self-report Walking Impairment Questionnaire (WIQ). Four studies measured QoL using the 36-Item Short Form Health Survey (SF-36) [26,27,29,34], while one study used the Vascular Quality of Life Questionnaire (VascuQoL) [31]. Four studies showed a significant improvement of functional status and QoL in the mHealth group [26,27,31,34], one study showed no effect [29], and two studies did not measure functional status or QoL [30,32].

**Table 2:** Characteristics of the study intervention and outcomes.

Study	Intervention	Comparator	Follow-up assessment	Outcomes
[34]	Referred to a local physical therapist; provided feedback using wearable activity tracker records	Received verbal walking advice and a brochure	Baseline, and 3, 6, 9, and 12 months	ACD^a^, FCD^b^: increased significantly in all groups (P<.01); WIQ^c^: total score improved in the SET^d^ group (P=.004); SF-36^e^: total score improved in the SET group (P<.001)
[31]	Daily activity goals setting using wearable activity monitor; provided feedback using wearable activity tracker records at each follow-up visit	Not reported	Baseline, and 3, 6, and 12 months	MWD^f^: increased significantly in mHealth^g^ group (P=.001); CD^h^: significantly improved in mHealth group (P=.005); VascuQoL^i^: significantly improved in mHealth group (P=.004)
[30]	Provided monthly feedback using wearable activity tracker records; electronic PAD^j^ book; weekly emails containing PAD tip; counseling and exercise prescriptions over the telephone	Received a hard copy of the PAD book	Baseline and 12 weeks	COT^k^, PWT^l^: increased significantly in mHealth group (P<.06); moderate-high activity minutes: increased significantly in mHealth group (P<.07)
[26]	Provided feedback (including new instructions) and motivation using wearable activity tracker records and logbook	Light-resistance exercise program group: 3 days/week	Baseline, and 1, 4, 8, and 12 weeks	COT, PWT, 6MWT^m^: increased significantly in mHealth group (P<.001); WIQ: increased significantly in mHealth group (P<.05); SF-36: increased significantly in mHealth group (P=.01)
[27]	Provided feedback (including new instructions) and motivation using wearable activity tracker records and logbook	Received verbal walking advice	Baseline, and 1, 2, 4, 6, 8, 10, and 12 weeks	COT, PWT: increased significantly in mHealth group (P<.001); WIQ: increased significantly in mHealth group (P<.05); SF-36: increased significantly in mHealth group (P<.01)
[29]	Provided coaching on exercise goals using wearable activity tracker records over the telephone; group telephone calls to share their successes and challenge experiences (2 calls/month)	Received no study intervention	Baseline, and 4.5 and 9 months	6MWT, WIQ, SF-36: no significant difference in mean change
[32]	Provided interactive wearable activity tracker and its online self-monitoring home page dashboard; bimonthly online video series	Bimonthly online video series	Baseline and 12 weeks	6MWT: increased significantly in mHealth group

^a^ACD: absolute claudication distance.

^b^FCD: functional claudication distance.

^c^WIQ: Walking Impairment Questionnaire.

^d^SET: supervised exercise therapy.

^e^SF-36: 36-Item Short Form Health Survey.

^f^MWD: maximum walking distance.

^g^mHealth: mobile health.

^h^CD: claudication distance.

^i^VascuQoL: Vascular Quality of Life Questionnaire.

^j^PAD: peripheral artery disease.

^k^COT: claudication onset time.

^l^PWT: peak walking time.

^m^6MWT: 6-minute walk test.

### Intervention Adherence and mHealth Functions

Table 3 summarizes the attrition and adherence rates, type of mHealth devices used, and applied mHealth functions.

The attrition rate in the mHealth groups ranged from 0% to 28%, and an adherence rate was reported in only two studies (exceeding 80% in both cases) [26,27]. Regarding the use of wearable activity trackers, in five studies the participants wore the trackers on their wrists [26,27,30,31,34], and in other studies they wore them on the ankles [29] or anterior thigh and waist [32]. The common functions applied to mHealth interventions were recording and display. In addition, the reminding/alerting function [31], guiding function [29], and communication function [32] were used.

**Table 3:** Summary of attrition and adherence rates, mobile health (mHealth) devices used, and applied mHealth functions.

Study	Attrition rate	Adherence rate	mHealth devices used	mHealth functions
[34]	mHealth-based SET^a^ group: 16%; SET group: 15%; control group: 18%	Not reported	Personal Activity Monitor accelerometer (PAM BV)	Recording, display
[31]	mHealth-based HBET^b^ group: 20%; control group: 24%	Not reported	Nike+ FuelBand (Nike, Inc)	Recording, display, reminder
[30]	mHealth-based HBET group: 0%; control group: 11%	Not reported	Fitbit Charge device (Fitbit, Inc)	Recording, display
[26]	mHealth-based HBET group: 12%; SET group: 13%; control group: 15%	mHealth-based HBET group: 81%; SET group: 82%	StepWatch3 (Orthoinnovations, Inc)	Recording, display
[27]	mHealth-based HBET group: 28%; SET group: 18%; control group: 23%	mHealth-based HBET group: 83%; SET group: 85%	StepWatch3 (Cyma Inc)	Recording, display
[29]	mHealth-based HBET group: 7%; control group: 7%	Not reported	Fitbit Zip (FitBit, Inc)	Recording, display, guide
[32]	No patients withdrew during the study period	Not reported	Gruve activity tracker (Gruve Solutions; Muve Inc); activPAL (PAL Technologies Ltd); online dashboard	Recording, display, communication (feedback)

^a^SET: supervised exercise therapy.

^b^HBET: home-based exercise therapy.

### Quality Assessment of Literature

**Figure 2:**
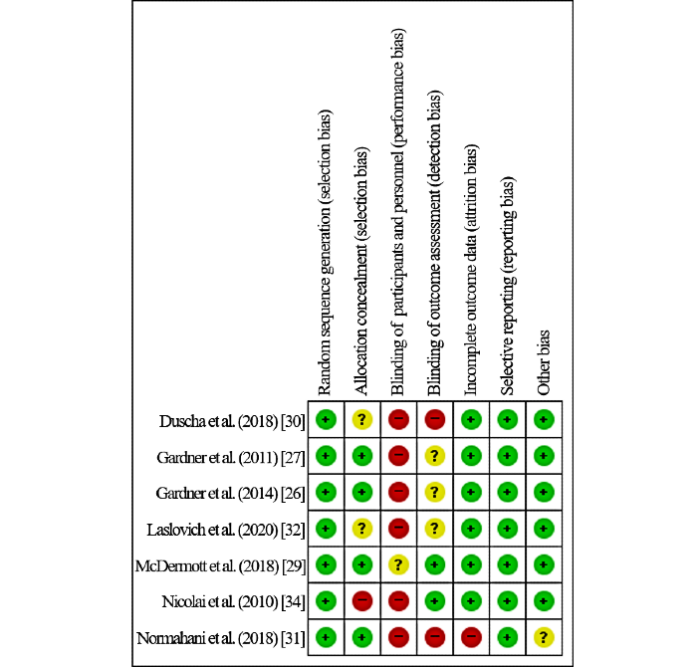
Risk of bias: individual studies.

**Figure 3:**
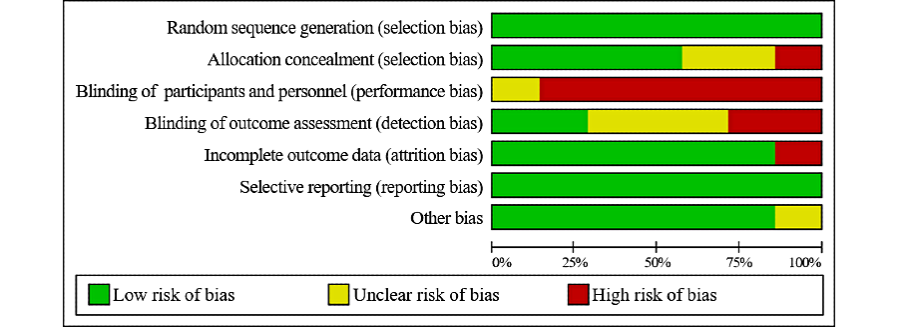
Risk of bias: summary graph.

The risk of bias analysis is shown in Figure 2 (individual studies) and Figure 3 (summary graph). All of the studies were assessed to have a low risk of selection bias. Due to maintaining the blinding of investigators and participants in exercise interventions, six of the seven studies were assessed to have a high risk of performance bias [26,27,30-32,34]. However, both attrition bias and reporting bias were generally assessed as low risk. With the exception of studies that reported that assessors were not blinded due to resource constraints [30,31], detection bias was assessed as unclear [26,27,32] or low risk [29,34].

### Meta-Analysis Findings: Effects of mHealth Exercise Interventions

#### Walking Performance

**Figure 4:**
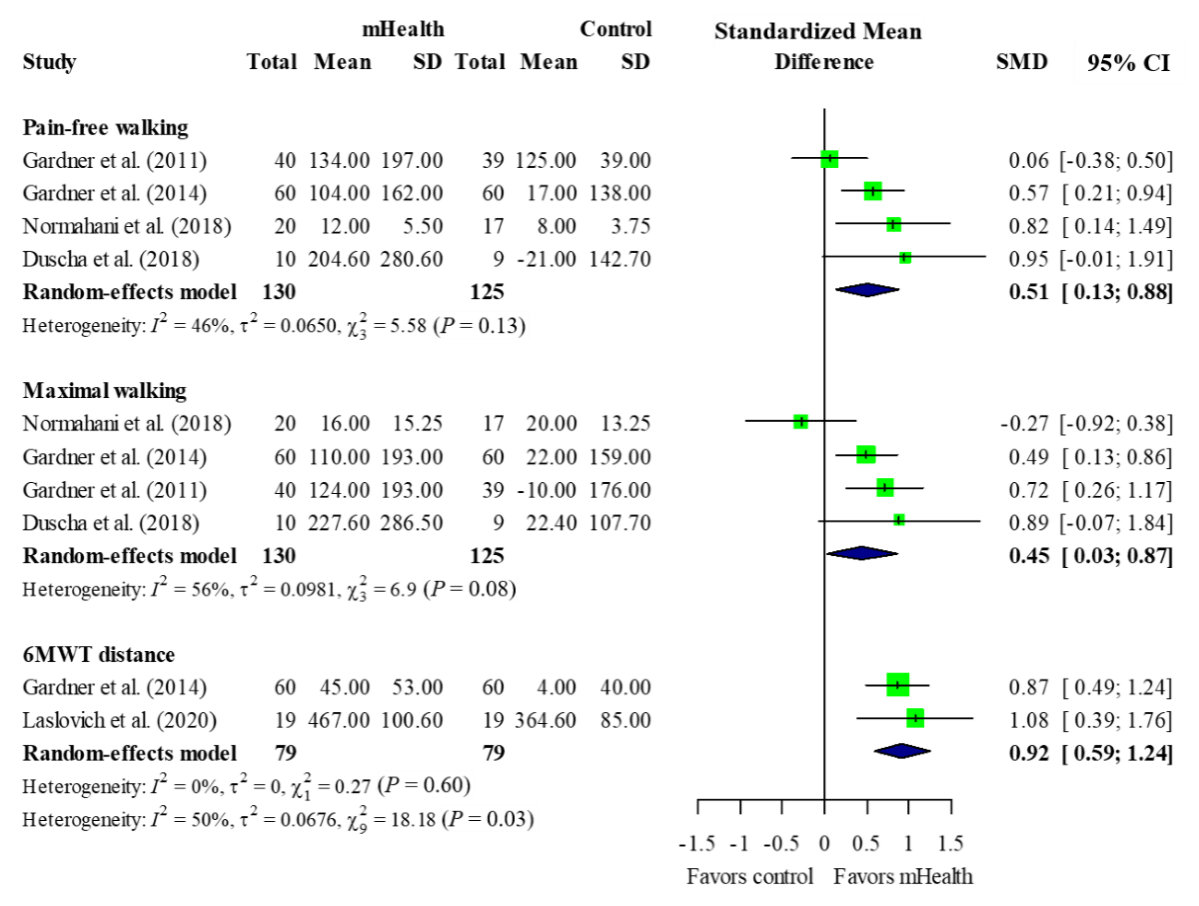
Forest plot of walking performance (up to the 12-week point). 6MWT: 6-minute walk test; mHealth: mobile health; SMD: standardized mean difference.

All six studies included in the meta-analysis reported walking performance. A treadmill test [26,27,30,31] and 6MWT [26,29,32] were used. The mHealth intervention groups showed an overall improvement in pain-free walking (SMD: 0.51, 95% CI 0.13-0.88; P=.008), maximal walking (SMD: 0.45, 95% CI 0.03-0.87; P=.04), and 6MWT distance (SMD: 0.92, 95% CI 0.59-1.24; P<.001) when compared with the control groups (Figure 4).

There was heterogeneity when pooling the pain-free walking (χ^2^=5.58, P=.13; *I*^2^=46%), maximal walking (χ^2^=6.9, P=.08; *I*^2^=56%), and 6MWT (χ^2^=0.27, P=.60; *I*^2^=0%) results. There was no significant difference found from omitting one single study using sensitivity analysis (Multimedia Appendix 3).

#### Functional Status

**Figure 5:**
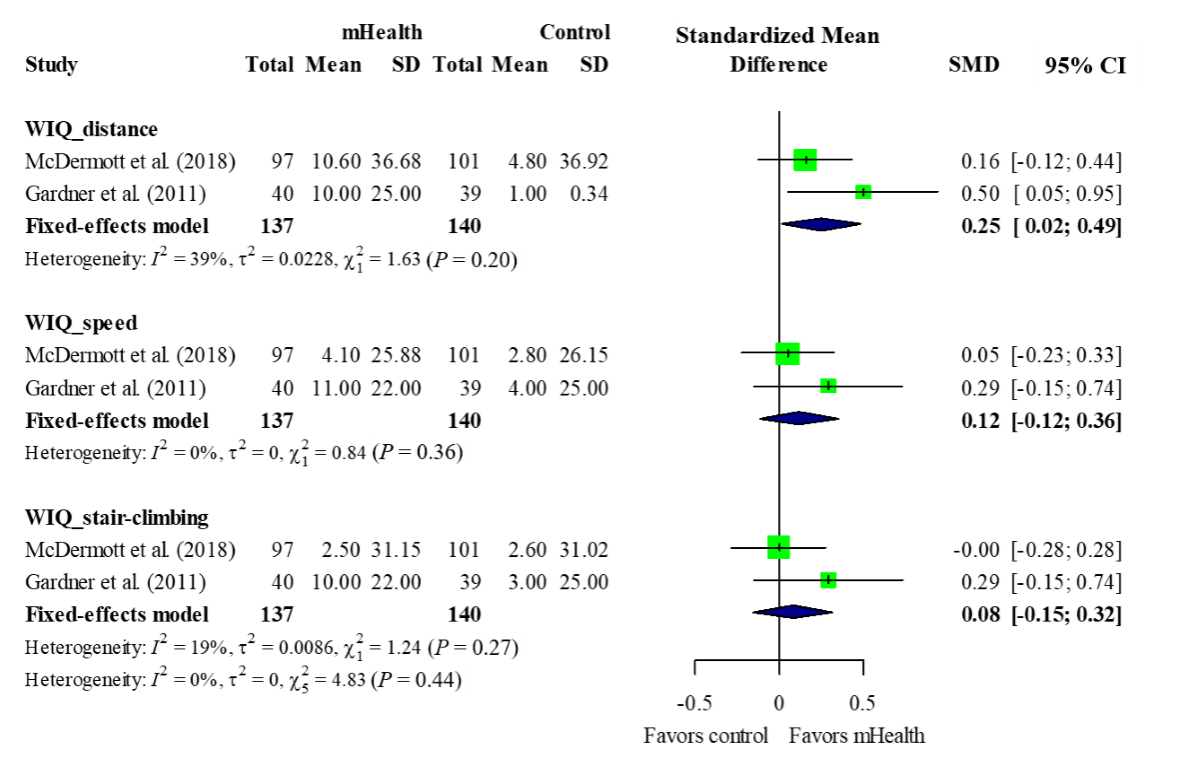
Forest plot of functional status. mHealth: mobile health; SMD: standardized mean difference; WIQ: Walking Impairment Questionnaire.

Reported functional status, two of which were included in our quantitative synthesis [27,29] (Figure 5).

The two included studies showed that the mHealth intervention groups significantly improved their WIQ distance (SMD: 0.25, 95% CI 0.02-0.49; P=.04) [27,29]. Other elements such as walking speed and stair-climbing ability did not show statistically significant improvements.

#### QoL

**Figure 6:**
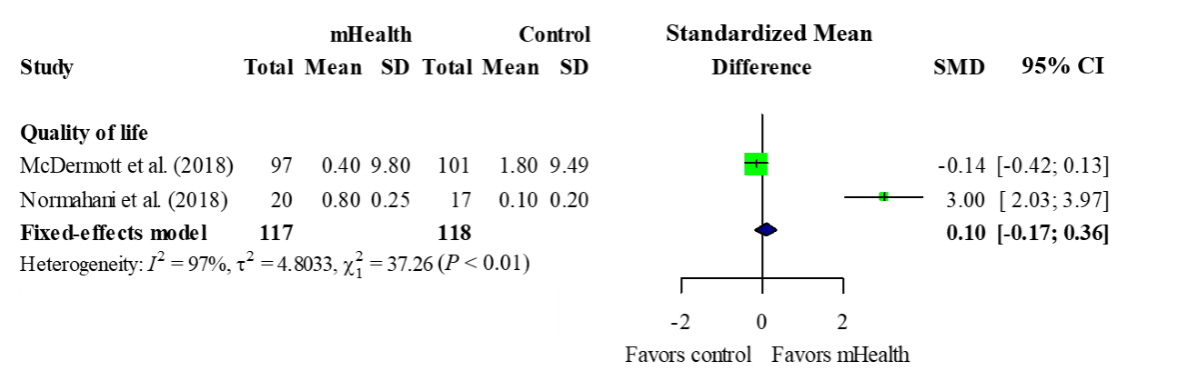
Forest plot of quality of life. mHealth: mobile health; SMD: standardized mean difference.

Four studies reported QoL, two [29,31] of which were included in the meta-analysis (Figure 6).

The two studies included in the meta-analysis measured QoL using VascuQoL [31] and SF-36 [29], respectively. The results showed that the mHealth intervention groups did not show significant improvements in QoL compared with the control groups (P=.48) but showed significant heterogeneity (χ^2^=37.3, P<.01; *I*^2^=97%).

## Discussion

### Principal Findings

In patients with PAD, effective and structured HBET can be a more accessible and acceptable alternative to SET in terms of burden and cost [3,8]. Therefore, this study is meaningful as the first meta-analysis of the effectiveness of applying mHealth as a strategy to provide structured exercise interventions at home for patients with PAD. In mHealth-based HBET, adherence is important for increasing the beneficial effects of exercise [8]. However, only two studies reported adherence rates [26,27]. This reflects the fact that while adherence in exercise intervention is a precursor and an important predictor for improvement of outcome, it is not always measured [35,36]. The adherence rates in the two above-mentioned studies both exceeded 80%. This is similar to the adherence rates reported in systematic reviews of exercise programs for older adults (65% to 86%) [37], RCTs featuring exercise referral schemes (pooled mean: 80%) [38], and exercise groups for patients with PAD (78%) [7]. Although maintaining high adherence rates is difficult in HBET [3], HBET using mHealth technologies such as wearable activity tracking devices seems to maintain high adherence. Future studies should report adherence rates; further, among the various measures of adherence [35,37,38], an appropriate and accurate measure for adherence rates for patients with PAD should be identified and applied.

A total of seven studies were included in our systematic review, and six studies were included in the meta-analysis. The studies included in the meta-analysis all applied mHealth techniques in HBET. During the 12-week interventions, the mHealth group showed significant improvements in pain-free walking, maximal walking, and 6MWT distance when compared with the control group. In one study that was not included in the pooled analysis of walking performance [29], the intervention was performed for 9 months and showed no effects on 6MWT distance in the mHealth group.

The functional status of the patients was measured using the WIQ, which has been validated as a measure of perceived difficulty concerning walking distances and speeds and the ability to climb stairs [39]; significant improvements were seen in walking distance but benefits regarding walking speed and stair-climbing ability were not observed. These results are similar to those of other exercise intervention studies, which reported significant effects on WIQ walking distance and no effects on other outcome measures [40,41].

The mHealth group did not show a significantly improved QoL. In other studies, the effect of mHealth-based interventions on QoL was unclear. Some meta-analyses have reported that mHealth-based interventions did not improve QoL in cancer survivors [42,43], while another study reported improved QoL in patients with coronary heart disease [44]. Therefore, the long-term effect of mHealth interventions on QoL should be investigated further.

### Limitations

A major limitation of this study was that the type of mHealth devices used in the reviewed studies was restricted to wearable activity trackers. It is important to monitor activity to promote physical activity in patients with PAD [31]; however, activity trackers may not have a sufficient impact in relation to inducing changes in health behaviors and improving adherence, as they provide a limited range of mHealth functions. Future studies need to investigate the effects of providing patients with PAD with a wider range of mHealth functions such as real-time advice and symptom monitoring [13,14,45], feedback and verification of achievement of individually set goals [6,13,14], and coaching chatbots [46]. In addition, this study was not able to review studies evaluating walking performance beyond 12 weeks, which was a result of the limited durations of the studies. Since all of the studies were based on structured exercise programs with recommended durations of at least 12 weeks [3], there were limited findings for long-term effects. PAD is a chronic disease for which treatment should involve sustained walking exercise; thus, it is important that interventions have effects on the long-term performance of walking exercises [3]. In future studies, the effects of long-term mHealth-based exercise interventions will need to be identified.

### Conclusions

This study provides evidence that mHealth-based exercise interventions applied through HBET for patients with PAD improve pain-free walking, maximal walking, 6MWT distance, and walking distance as elements of functional status. In addition, an HBET group that received the mHealth intervention showed an adherence rate similar to the SET group. We found that using mHealth as part of exercise interventions is an important strategy to improve the walking ability and exercise adherence rate of patients with PAD at home or in the community in their daily living environments. Future studies should consider the use of various and suitable functions of mHealth to improve the adherence rate and the effectiveness of exercise interventions.

## Appendix

Multimedia Appendix 1 PRISMA (Preferred Reporting Items for Systematic Reviews and Meta-Analyses) checklist.

Multimedia Appendix 2 Search strategies.

Multimedia Appendix 3 Sensitivity analyses of pooled effect estimates.

## Abbreviations

6MWT: 6-minute walk test
HBET: home-based exercise therapy
mHealth: mobile health
PRISMA: Preferred Reporting Items for Systematic Reviews and Meta-Analyses
PWT: peak walking time
QoL: quality of life
PAD: peripheral artery disease
RCT: randomized controlled trial
SET: supervised exercise therapy
SF-36: 36-Item Short Form Health Survey
SMD: standardized mean difference
VascuQoL: Vascular Quality of Life Questionnaire
WIQ: Walking Impairment Questionnaire
