# Supplementary material for: Effectiveness of Mobile Health–Based Exercise Interventions for Patients with Peripheral Artery Disease: Systematic Review and Meta-Analysis
Source: JMIR Mhealth Uhealth. 2021 Feb 15;9(2):e24080. doi: 10.2196/24080 (PMC7920758; doi:10.2196/24080)
Supplement: Multimedia Appendix 2 [file mhealth_v9i2e24080_app2.docx]

Multimedia Appendix 2. Search strategies.

Search Date: December 4, 2020

| 1. Database: PubMed | | | | |
| --- | --- | --- | --- | --- |
| PICO | Keyword | Search | Query | Result |
| P | peripheral arterial disease | #1 | "Peripheral Arterial Disease"[Mesh] OR "peripheral arterial disease" OR "peripheral artery disease" OR "peripheral arterial obstructive disease" OR "peripheral occlusive disease" OR "peripheral vascular disease" OR "peripheral vascular diseases" OR claudication | 46,282 |
| I | mHealth | #2 | "Telemedicine"[Mesh] OR mHealth OR "m Health" OR "mobile health" | 50,867 |
| I | mHealth: devices | #3 | "Smartphone"[Mesh] OR smartphone OR smartphones OR "smart phone" OR mobilephone OR "mobile phone" OR mobilephones OR "mobile phones" OR "Mobile Applications"[Mesh] OR "mobile application" OR "mobile applications" OR "mobile apps" OR "mobile app" OR "mobile health application" OR "Wearable Electronic Devices"[MeSH Terms] OR "wearable device" OR "wearable devices" OR wearables OR wearable OR "Fitness Trackers"[Mesh] OR "activity tracker" OR "activity trackers" OR "activity monitor" OR "activity monitors" | 53,643 |
|  |  |  | #1 AND (#2 OR #3) | 101 |

| 2. Database: CINAHL with Fulltext | | | | |
| --- | --- | --- | --- | --- |
| PICO | Keyword | Search | Query | Result |
| P | peripheral arterial disease | #1 | (MH "Peripheral Vascular Diseases+") OR "peripheral arterial disease" OR "peripheral artery disease" OR "peripheral arterial obstructive disease" OR "peripheral occlusive disease" OR "peripheral vascular disease" OR "peripheral vascular diseases" OR claudication | 22,673 |
| I | mHealth | #2 | (MH "Telehealth+") OR mHealth OR "m Health" OR "mobile health" | 29,384 |
| I | mHealth: devices | #3 | (MH "Smartphone") OR smartphone OR smartphones OR "smart phone" OR mobilephone OR "mobile phone" OR mobilephones OR "mobile phones" OR (MM "Mobile Applications") OR "mobile application" OR "mobile applications" OR "mobile health application" OR "mobile apps" OR "mobile app" OR "wearable device" OR "wearable devices" OR wearables OR wearable OR "activity tracker" OR "activity trackers" OR "activity monitor" OR "activity monitors" | 22,973 |
|  |  |  | #1 AND (#2 OR #3) | 130 |

| 3. Database: Cochrane CENTRAL | | | | |
| --- | --- | --- | --- | --- |
| PICO | Keyword | Search | Query | Result |
| P | peripheral arterial disease | #1 | MeSH descriptor: [Peripheral Arterial Disease] explode all trees OR "peripheral arterial disease" OR "peripheral artery disease" OR "peripheral arterial obstructive disease" OR "peripheral occlusive disease" OR "peripheral vascular disease" OR "peripheral vascular diseases" OR claudication | 6,545 |
| I | mHealth | #2 | [Telemedicine] explode all trees OR mHealth OR "m Health" OR "mobile health" | 8,996 |
| I | mHealth: devices | #3 | [Smartphone] explode all trees OR smartphone OR smartphones OR "smart phone" OR mobilephone OR "mobile phone" OR mobilephones OR "mobile phones" OR [Mobile Applications] explode all trees OR "mobile application" OR "mobile applications" OR "mobile health application" OR "mobile apps" OR "mobile app" OR [Wearable Electronic Devices] explode all trees OR "wearable device" OR "wearable devices" OR wearables OR wearable OR MeSH descriptor: [Fitness Trackers] explode all trees OR "activity tracker" OR "activity trackers" OR "activity monitor" OR "activity monitors" | 10,481 |
|  |  |  | #1 AND (#2 OR #3) | 268 |

| 4. Database: Embase | | | | |
| --- | --- | --- | --- | --- |
| PICO | Keyword | Search | Query | Result |
| P | peripheral arterial disease | #1 | 'peripheral occlusive artery disease'/exp OR 'peripheral arterial disease' OR 'peripheral artery disease' OR 'peripheral arterial obstructive disease' OR 'peripheral occlusive disease' OR 'peripheral vascular disease' OR 'peripheral vascular diseases' OR claudication | 216,479 |
| I | mHealth | #2 | mHealth OR 'm Health' OR 'mobile health' | 14,114 |
| I | mHealth: devices | #3 | 'smartphone'/exp OR smartphone OR smartphones OR 'smart phone' OR mobilephone OR 'mobile phone' OR mobilephones OR 'mobile phones' OR 'mobile application'/exp OR 'mobile health application'/exp OR 'mobile applications' OR 'mobile health application' OR 'mobile application' OR 'mobile apps' OR 'mobile app' OR 'wearable device'/exp OR 'wearable device' OR 'wearable devices' OR wearables OR wearable OR 'activity monitor'/exp OR 'activity tracker' OR 'activity trackers' OR 'activity monitor' OR 'activity monitors' | 65,238 |
|  |  |  | #1 AND (#2 OR #3) | 208 |

| 5. Database: IEEE Xplore Digital Library | | | | |
| --- | --- | --- | --- | --- |
| PICO | Keyword | Search | Query | Result |
| P | peripheral arterial disease | #1 | "peripheral arterial disease" OR "peripheral artery disease" OR "peripheral vascular disease" OR "peripheral vascular diseases" OR claudication | 116 |
| I | mHealth | #2 | "Telemedicine" OR mHealth OR "m Health" OR "mobile health" | 9,623 |
| I | mHealth: devices | #3 | "Smartphone" OR smartphones OR "smart phone" OR "mobile phone" OR mobilephones OR "mobile phones" OR "mobile application" OR "mobile applications" OR "mobile apps" OR "mobile app" OR wearable OR "Fitness Trackers" OR "activity tracker" OR "activity trackers" OR "activity monitor" OR "activity monitors | 63,372 |
|  |  |  | #1 AND (#2 OR #3) | 9 |

| 6. Database: Web of science | | | | |
| --- | --- | --- | --- | --- |
| PICO | Keyword | Search | Query | Result |
| P | peripheral arterial disease | #1 | "peripheral arterial disease" OR "peripheral artery disease" OR "peripheral arterial obstructive disease" OR "peripheral occlusive disease" OR "peripheral vascular disease" OR "peripheral vascular diseases" OR claudication | 35,134 |
| I | mHealth | #2 | "Telemedicine" OR mHealth OR "m Health" OR "mobile health" | 30,833 |
| I | mHealth: devices | #3 | smartphone OR smartphones OR "smart phone" OR mobilephone OR "mobile phone" OR mobilephones OR "mobile phones" OR "mobile application" OR "mobile applications" OR "mobile apps" OR "mobile app" OR "mobile health application" OR "Wearable Electronic Devices" OR "wearable device" OR "wearable devices" OR wearables OR wearable OR "Fitness Trackers" OR "activity tracker" OR "activity trackers" OR "activity monitor" OR "activity monitors" | 85,681 |
|  |  |  | #1 AND (#2 OR #3) | 96 |

| 7. Database: Scopus | | | |  |
| --- | --- | --- | --- | --- |
| PICO | Keyword | Search | Query | Result |
| P | peripheral arterial disease | #1 | "peripheral arterial disease" OR "peripheral artery disease" OR "peripheral arterial obstructive disease" OR "peripheral occlusive disease" OR "peripheral vascular disease" OR claudication | 92,644 |
| I | mHealth | #2 | Telemedicine OR mHealth OR "m Health" OR "mobile health" | 81,203 |
| I | mHealth: devices | #3 | smartphone OR "smart phone" OR mobilephone OR "mobile phone" OR "mobile application" OR "mobile app" OR "mobile health application" OR "Wearable Electronic Devices" OR "wearable device" OR wearable OR "Fitness Trackers" OR "activity tracker" OR "activity monitor" | 248,801 |
|  |  |  | #1 AND (#2 OR #3) | 676 |
