# Supplementary material for: Effectiveness of Mobile Health–Based Exercise Interventions for Patients with Peripheral Artery Disease: Systematic Review and Meta-Analysis
Source: JMIR Mhealth Uhealth. 2021 Feb 15;9(2):e24080. doi: 10.2196/24080 (PMC7920758; doi:10.2196/24080)

Multimedia Appendix 3. Sensitivity analyses of pooled effect estimates.

1. Pain-free walking

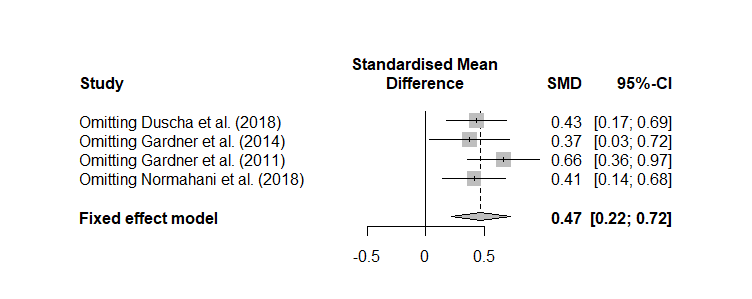

2. Maximal walking


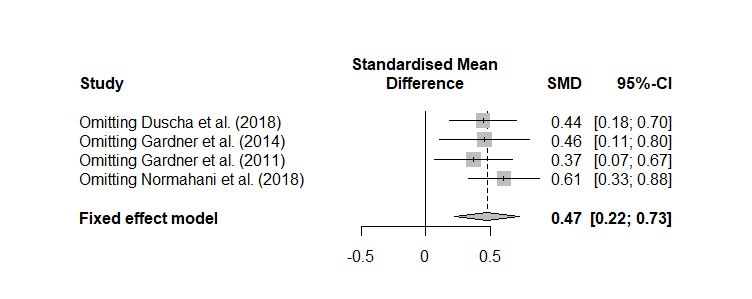


1. 6MWT


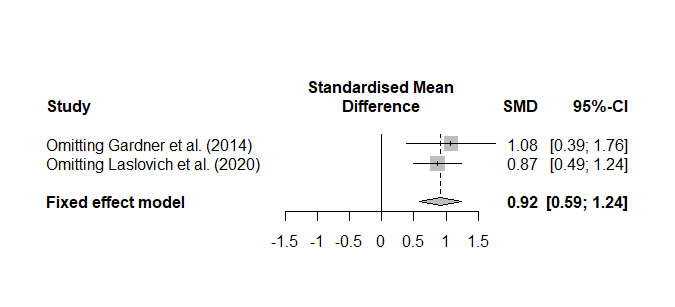

Supplement: Multimedia Appendix 3 [file mhealth_v9i2e24080_app3.docx]
